# EZH2 is a sensitive marker of malignancy in salivary gland tumors

**DOI:** 10.1186/s13000-015-0392-z

**Published:** 2015-09-17

**Authors:** Szofia Hajósi-Kalcakosz, Eszter Vincze, Katalin Dezső, Sándor Paku, András Rókusz, Zoltán Sápi, Erika Tóth, Péter Nagy

**Affiliations:** First Department of Pathology and Experimental Cancer Research, Semmelweis University, Üllői út 26, Budapest, H-1085 Hungary; Heim Pál Children’s Hospital, Budapest, Hungary; Tumor Progression Research Group, Joint Research Organization of the Hungarian Academy of Sciences and Semmelweis University, Budapest, Hungary; Pathology Department, National Institute of Oncology, Budapest, Hungary

## Abstract

**Background:**

The immunohistochemical detection of Enhancer of zeste homologue 2 (EZH2) proved to be a useful tool to recognize the malignant nature of tumors in a wide variety of neoplasms. The histological diagnostics of salivary gland tumors is a challenging task, and a reliable marker of malignancy would be extremely helpful.

**Methods:**

EZH2 expression was investigated in 54 malignant and 40 benign salivary gland tumors of various histological types by standard immunohistochemistry.

**Results:**

The majority (*n* = 52) of the malignant tumors stained positively, while all the investigated benign tumors were negative for EZH2.

**Conclusions:**

EZH2 expression in salivary gland tumors, similarly to the tumors of other organs is not characteristic for any tumor type, but is a solid marker of the malignant nature of the tumors.

**Electronic supplementary material:**

The online version of this article (doi:10.1186/s13000-015-0392-z) contains supplementary material, which is available to authorized users.

## Background

Although tumors of the salivary glands are not among the most common neoplasms, they provide a serious challenge for pathologists. The multitude of tumor types and overlapping histological features often give rise to multiple interpretations. Moreover, on occasion it can be problematic to distinguish between the benign and malignant nature of certain specimens. Of course, ancillary techniques are widely used. Certain molecular lesions have been recognized in different tumors and immunohistochemistry is applied on an everyday routine basis [1–3]. However, these techniques provide only limited help and the diagnosis is mostly determined by the H&E stained sections.

Enhancer of zeste homologue 2 (EZH2) is a widely studied histone methyl transferase [4, 5]. Recently we have reported that the immunohistochemical examination of this protein can effectively distinguish between benign and malignant hepatic tumors [6]. Similar observations have been made in intraductal papillary neoplasms of bile ducts [7], effusion cytology [8], etc. To our knowledge, there is quite limited information on the expression of this potential tumor marker in salivary gland tumors. The available data, however, suggest that there may be a connection between the behavior of salivary gland tumors and EZH2 expression as well [9, 10]. The purpose of our study is to examine EZH2 expression in a variety of benign and malignant salivary gland tumors, and to investigate if it provides any useful information for their recognition. All 40 benign salivary gland tumors investigated were negative for EZH2, while 52 of the 54 malignant tumors proved to be positive. Based on this observation EZH2 immunohistochemistry might provide valuable information for the histological examination of salivary gland tumors.

## Methods

We selected 54 (Additional file 1: Table S1) malignant and 40 benign (Additional file 2: Table S2) salivary gland tumors from the archives of the First Department of Pathology and Experimental Cancer Research, Semmelweis University (Budapest, Hungary) and Pathology Department of National Institute of Oncology (Budapest, Hungary). The study was approved by the ethics committee of the Semmelweis University. The tumors were diagnosed according to standard diagnostic criteria and immunohistochemical staining. Formalin-fixed paraffin-embedded tissue was used for the immunohistochemical reactions. Staining was performed using an automated Leica Bond immunostainer, with the Leica Bond Polymer refine detection system and 3,3′ Diaminobenzidine (DAB) as the chromogen. Antigen retrieval was achieved with Bond Epitope Retrieval Solution 2 (high pH) for 20 min. The primary antibody was a mouse monoclonal anti-EZH2 (clone 11/EZH2) from BD Biosciences (San Jose CA, USA) (dilution 1:100). The reaction resulted in nuclear staining. Scores were assigned based on the density of positivity by using negative (score =0, < 5 % of nuclei staining); weak (score = 1, 5–10 % of nuclei staining); moderate (score = 2, 11–50 % of nuclei staining); and strong (score = 3; >50 % of nuclei staining).

## Results

EZH2 immunohistochemical staining resulted in a clear nuclear reaction. The non-tumorous portion of the salivary gland tissue always remained negative. The lymphoid cells were frequently positive, as well as the nuclei of the squamous epithelium on occasional mucosal pieces on the tissue.

### Benign tumors

**Fig. 1:**
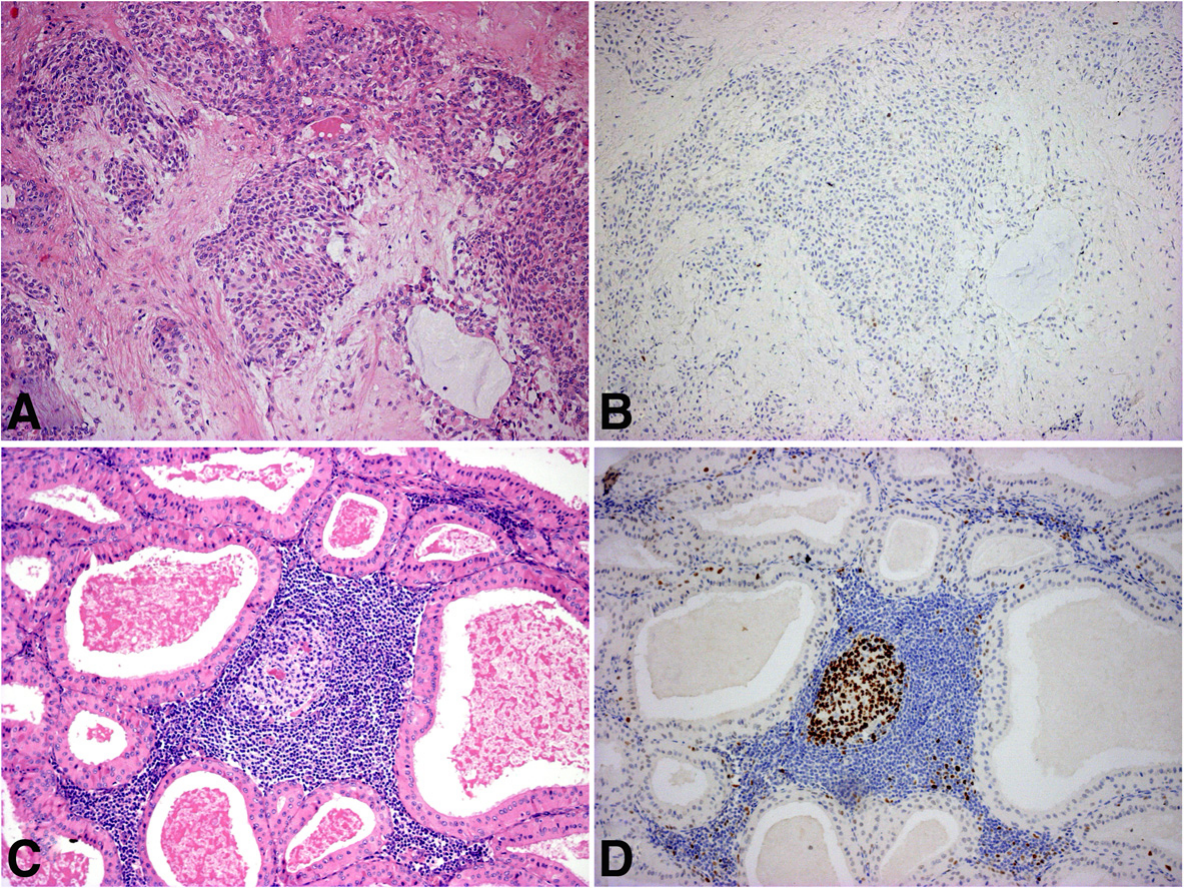
H & E (**a**) and EZH2 staining (**b**) of a pleiomorphic adenoma (40x); there are only scattered positive nuclei in all components of the tumor; H & E (**c**) and EZH2 staining (**d**) of a Warthin tumor (40x); the epithelial component of the tumor is negative, while the lymphocytes are frequently positive for EZH2

The 18 investigated pleiomorphic adenomas (Fig. 1a and b) included a wide range of this highly variable tumor type. Two of the specimens were recurrent multifocal tumors, but regardless of the actual structure, were consistently negative for EZH2. Occasionally scattered EZH2 positive nuclei were present in the epithelial component, but the ratio of these marked nuclei was always under 5 %.

The epithelial components of the Warthin tumors (Fig. 1c and d) were also consistently negative, while the lymphoid follicles, especially the germinative centers, were stained for EZH2 similar to the normal lymphoid tissue. Thus, these tumors were regarded as negative, not unlike other less common benign tumors such as basal cell adenomas, oncocytomas and cystadenomas.

### Malignant tumors

**Fig. 2:**
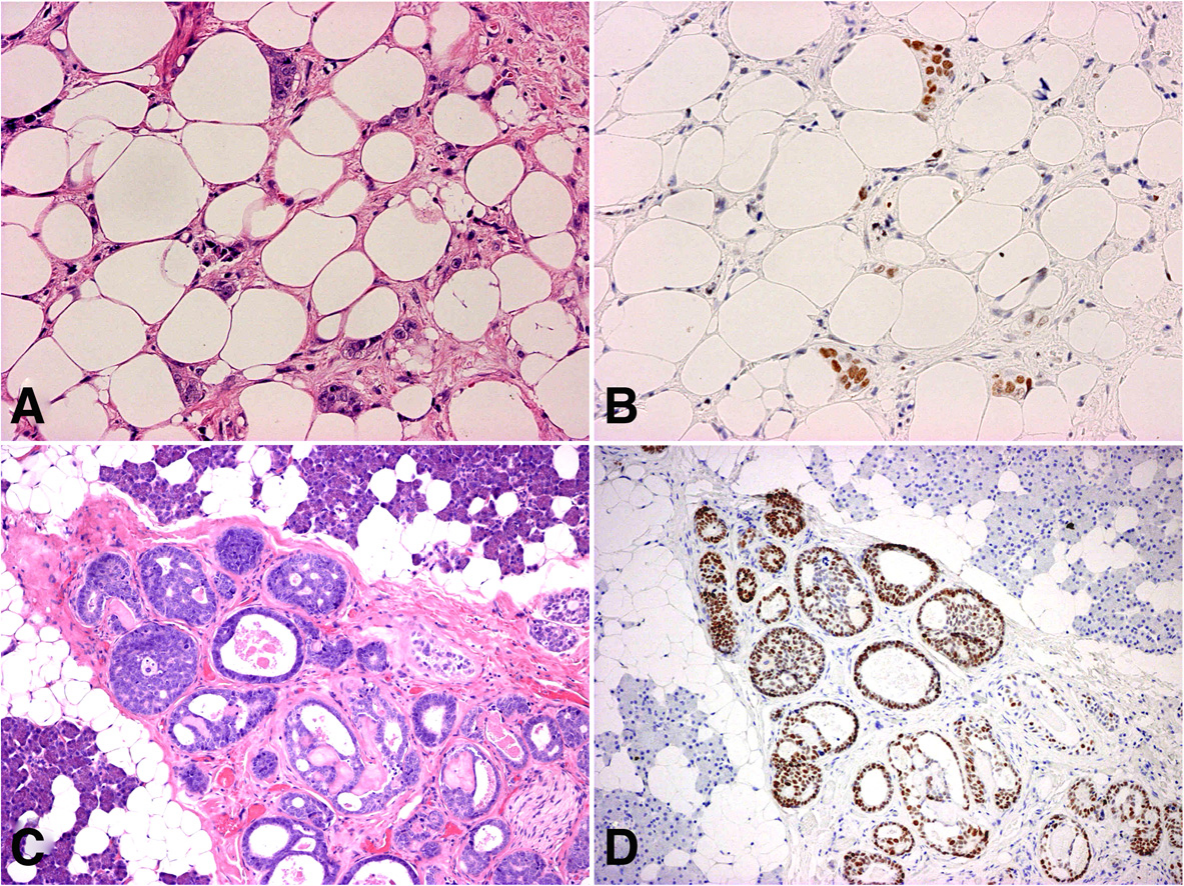
H & E (**a**) and EZH2 staining (**b**) of the fat tissue around a mucoepidermoid carcinoma (80x); the EZH2 reaction nicely reveals the scattered tumor cells; H & E (**c**) and EZH2 staining (**d**) of an adenoid cystic carcinoma (80x); EZH2 positive nuclei are common

Mucoepidermoid carcinoma (Fig. 2a and b) is the most common malignant tumor of the salivary glands. This tumor type was represented in the highest frequency (*n* = 17) of all the tumors studied. All but one of the investigated tumors stained positively for EZH2. The negative tumor was a small preoperative excision. Since EZH2 expression is mostly focal in the tumors, this negative result may be the consequence of a sampling error. Immunostaining was usually more extensive in the epidermoid component. No reliable relationship could be observed with tumor grade, but poorly differentiated components with infiltrative growth pattern were also positive.

All the studied adenoid cystic carcinomas (*n* = 13) stained positively with EZH2 antibody (Fig. 2c and d). The growth pattern of the tumor had no influence on the staining. Preferential staining of the abluminal cells could be observed in a few tumors, but this feature was not consistent. Perineural invading components of the tumors were also positive.

**Fig. 3:**
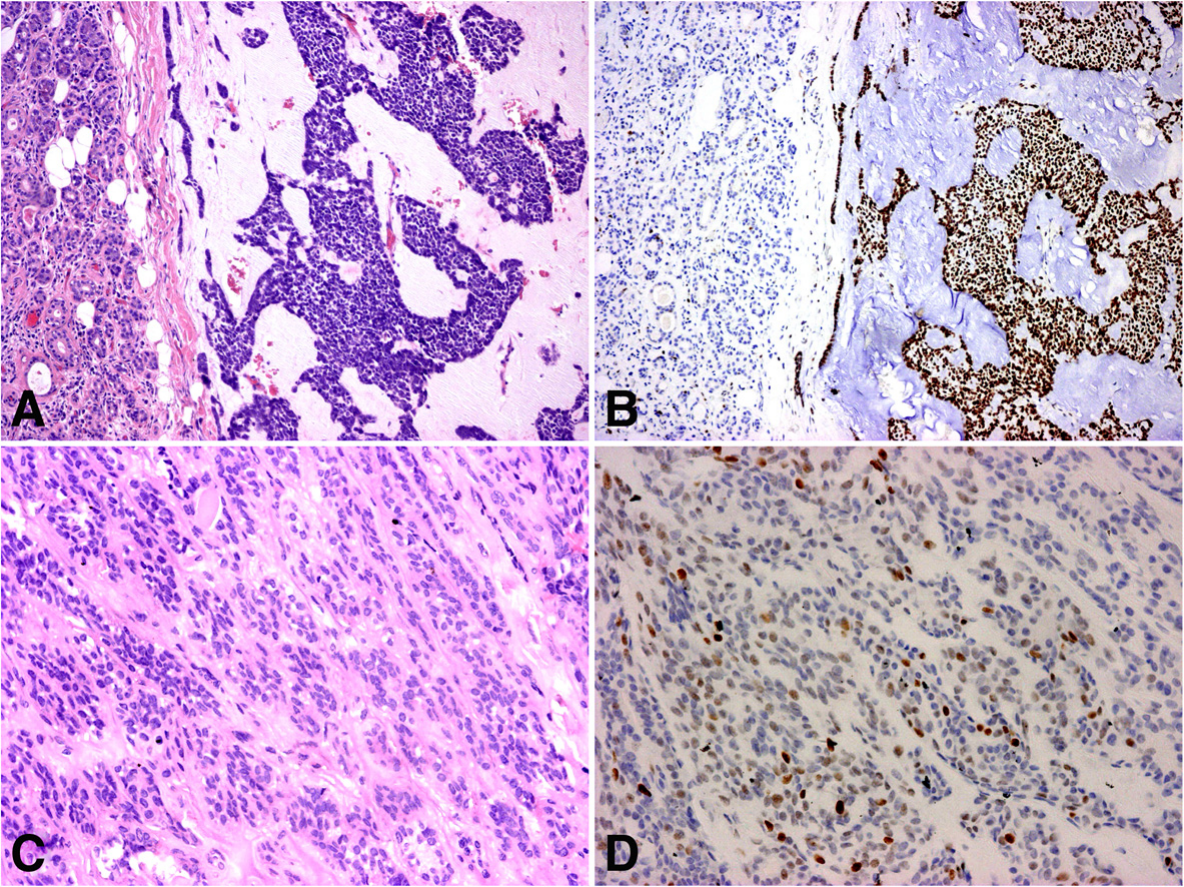
H & E (**a**) and EZH2 staining (**b**) of a poorly differentiated carcinoma ex pleiomorphic adenoma (40x); the tumor is strongly positive, the surrounding normal salivary gland remains negative; H & E (**c**) and EZH2 staining (**d**) of a polymorphous low-grade adenocarcinoma (80x); there is a heterogeneous staining for EZH2, but more than 20 % of the nuclei are positive

Eight carcinoma ex pleiomorphic adenoma were included. All were adenocarcinomas: one with focal squamous differentiation, one with dominant adenoid cystic carcinoma and another with malignant myoepithelioma components. Regardless of the histological structure, they were positive for EZH2 (Fig. 3a and b). One of the sections contained a preexistent adenoma which remained negative. The lymph node metastasis of a poorly differentiated adenocarcinoma was also positive.

Four of the acinic cell carcinomas (*n* = 5) had microcystic growth pattern, with one showing occasional papillary structures. All were variably positive. The single solid acinic cell carcinoma had, however, very few positively stained nuclei which was below our 5 % threshold. The three polymorphous low grade adenocarcinomas stained positively (Fig. 3c and d).

In case of two myoepithelial and one basal cell tumors, the malignant diagnosis was based on the high mitotic rate or invasive growth pattern. The preexistent diagnoses were nicely supported by the positive EZH2 staining. Three non-classifiable adenocarcinomas, one squamous cells carcinoma and one clear cell carcinoma also stained positively.

## Discussion

We have investigated the expression of EZH2 by immunohistochemistry on the most common types of salivary gland tumors. All the benign tumors (*n* = 40) were negative, but the majority of the malignant tumors (52/54) regardless of the histological type proved to be positive. EZH2 is the catalytic subunit of polycomb repressive complex 2 (PRC2). It catalyzes the trimethylation of lysine 27 on histone H3 (H3K27me3), and mediates transcriptional silencing [4, 5]. Gain of function mutations of EZH2 represent a promising therapeutic target in germinal center lymphomas [11], and increased expression of this protein has been reported in several other tumors [12–19]. It is usually an unfavorable prognostic marker. We have described that EZH2 is expressed in most of the malignant liver tumors, but it is absent from the benign tumors and reactive proliferative lesions [6]. This reaction was useful to recognize the malignant behavior of intraductal papillary neoplasms of bile ducts [7], hepatic and pancreatic cystic neoplasms [20] and squamous cell tumors of the skin [21]. EZH2 proved to be a unique marker of malignancy in effusion cytology [8]. Such a reliable marker of malignant tumors would be extremely helpful for salivary gland tumors as well due to their diverse histological structure, and the common morphological overlap between benign and malignant tumors. Assessment of proliferative activity [22] and several other markers (Human α-defensin, maspin, RB1-inducible coiled-coil 1, etc.) [2, 23–25] has been proposed to address this problem. High expression of EZH2 detected by immunohistochemistry has been reported to predict poor survival for patients with adenoid cystic carcinoma [9]. Increased EZH2 staining was more common in malignant myoepithelial tumors [10]. High expression of H3K9me3 is a strong predictor of poor survival in patients with salivary adenoid cystic carcinoma [26]. Although this DNA modification is related with another enzyme, it may indicate that the activity of histone methylation might be connected with the biological behavior of salivary gland tumors. As far as we know, our study is the first to involve a comprehensive set of salivary gland tumors. Our finding is comparable to the result found on other types of tumors. EZH2 is a highly sensitive (96.3 %) and specific (100 %) indicator of malignancy in salivary gland tumors. According to our results its positive predictive value is 100 %, while its negative predictive value is 95.24 %. The increased expression of this enzyme cannot be associated with any specific cell type, and so, similarly to the tumors of other organs, it does not provide any help for the histological classification of the tumors. Our sample size for the individual tumor types is too low to search for connection between the level of overexpression and prognosis. It is also crucial to investigate large series of tumors (e.g. basal cell adenoma v.s. carcinoma, myoepithelial, oncocytic tumors) where there is a broad gray border zone between benign and malignant tumors making the distinction between them highly challenging. Our result indicates that it would be worth performing such studies along with elucidating the molecular mechanism of EZH2 upregulation in salivary gland tumors. Such studies are most advanced in case of follicular lymphomas, where EZH2 has become a potential target of therapeutic approaches [11].

Interestingly, increased EZH2 expression has been reported in oral squamous cell carcinomas compared to dysplastic and normal epithelium [27]. The judgment of the squamous epithelium is another shaky field of oral pathology. In our slides the accidental non tumorous epithelium was always strongly decorated by the EZH2 antibody.

## Conclusion

In conclusion, the majority of malignant salivary gland tumors is EZH2 positive. This immunohistochemical reaction may be a useful tool to recognize them; however, it does not help to distinguish between different varieties of salivary gland carcinomas.

## Additional files

Additional file 1: Table S1.
**Malignant tumors.** (DOCX 14 kb) Additional file 2: Table S2.
**Benign tumors.** (DOCX 13 kb)
