# Supplementary material for: EZH2 is a sensitive marker of malignancy in salivary gland tumors
Source: Diagn Pathol. 2015 Sep 17;10:163. doi: 10.1186/s13000-015-0392-z (PMC4574143; doi:10.1186/s13000-015-0392-z)
Supplement: Additional file 1: Table S1. — Malignant tumors. (DOCX 14 kb) [file 13000_2015_392_MOESM1_ESM.docx]

Additional file 1: Table S1. Malignant tumors

| **Tumor type** | **Age** | **Gender (f/m)** | **Immunohistochemical staining score** | | | | | | |
| --- | --- | --- | --- | --- | --- | --- | --- | --- | --- |
|  |  |  | **Negative** | **Positive (+)** | | **Positive (++)** | | **Positive (+++)** | |
| **Mucoepidermoid carcinoma (17)** | 17-77 | 7/10 | 1 | | 10 | | 5 | | 1 |
| **Adenoid cystic carcinoma (13)** | 21-85 | 9/4 | 0 | | 6 | | 2 | | 5 |
| **Carcinoma ex pleiomorphic adenoma (8)** | 41-81 | 5/3 | 0 | | 3 | | 3 | | 2 |
| **Acinic cell carcinoma (5)** | 20-57 | 3/2 | 1 | | 3 | | 1 | | 0 |
| **PLGACC (3)** | 51-64 | 1/2 | 0 | | 3 | | 0 | | 0 |
| **Others (8)** | 29-68 | 4/4 | 0 | | 2 | | 3 | | 3 |

Scores were assigned based on the density of nuclear positivity by using negative (score =0, < 5% of nuclei staining); weak (score=1, 5-10% of nuclei staining); moderate (score=2, 11-50% of nuclei staining); and strong (score=3; >50% of nuclei staining).

PLGACC: Polymorphous low grade adenocarcinoma
