# Supplementary material for: EZH2 is a sensitive marker of malignancy in salivary gland tumors
Source: Diagn Pathol. 2015 Sep 17;10:163. doi: 10.1186/s13000-015-0392-z (PMC4574143; doi:10.1186/s13000-015-0392-z)
Supplement: Additional file 2: Table S2. — Benign tumors. (DOCX 13 kb) [file 13000_2015_392_MOESM2_ESM.docx]

Additional file 2: Table S2. Benign tumors

| **Tumor type** | **Age** | **Gender (f/m)** | **Negative nuclear staining** |
| --- | --- | --- | --- |
| **Warthin tumor (13)** | 40-77 | 7/6 | 13 |
| **Pleiomorphic adenoma (18)** | 17-72 | 12/6 | 18 |
| **Others (9)** | 34-77 | 6/3 | 9 |

Scores were assigned based on the density of nuclear positivity by using negative (score =0, < 5% of nuclei staining); weak (score=1, 5-10% of nuclei staining); moderate (score=2, 11-50% of nuclei staining); and strong (score=3; >50% of nuclei staining).
